# Swimming against the tide: investigations of the C-bouton synapse

**DOI:** 10.3389/fncir.2014.00106

**Published:** 2014-09-18

**Authors:** Adam S. Deardorff, Shannon H. Romer, Patrick M. Sonner, Robert E. W. Fyffe

**Affiliations:** Boonshoft School of Medicine, Department of Neuroscience, Cell Biology and Physiology, Wright State UniversityDayton, OH, USA

**Keywords:** SK, Kv2.1, α-motoneuron, acetylcholine, C-boutons, afterhyperpolarization, subsurface cistern

## Abstract

C-boutons are important cholinergic modulatory loci for state-dependent alterations in motoneuron firing rate. m2 receptors are concentrated postsynaptic to C-boutons, and m2 receptor activation increases motoneuron excitability by reducing the action potential afterhyperpolarization. Here, using an intensive review of the current literature as well as data from our laboratory, we illustrate that C-bouton postsynaptic sites comprise a unique structural/functional domain containing appropriate cellular machinery (a “signaling ensemble”) for cholinergic regulation of outward K^+^ currents. Moreover, synaptic reorganization at these critical sites has been observed in a variety of pathologic states. Yet despite recent advances, there are still great challenges for understanding the role of C-bouton regulation and dysregulation in human health and disease. The development of new therapeutic interventions for devastating neurological conditions will rely on a complete understanding of the molecular mechanisms that underlie these complex synapses. Therefore, to close this review, we propose a comprehensive hypothetical mechanism for the cholinergic modification of α-MN excitability at C-bouton synapses, based on findings in several well-characterized neuronal systems.

## INTRODUCTION

The neuromuscular system provides rapid and coordinated force generation, whereby the number and firing rate of recruited motor units are systematically adjusted to meet environmental demands (Monster and Chan, 1977; Henneman and Mendell, 1981; Clamann, 1993; Cope and Sokoloff, 1999). Indeed, the elegant simplicity with which animals navigate their environment relies on neural circuitry that is inherently modifiable, and the ability to perform a variety of motor tasks while responding quickly to unexpected perturbations and threats is essential for individual survival (Ladle et al., 2007; Miri et al., 2013). Control of α-MN repetitive firing properties is a therefore highly conserved and critical adaption of mammalian and non-mammalian species alike, and identifying the responsible spinal circuits has been of essential importance in our understanding of neuromuscular function and dysfunction (Miles and Sillar, 2011).

For more than 50 years, a particular class of synapse in the spinal cord ventral horn – the C-bouton – has generated sustained interest among α-MN anatomists and physiologists. Unambiguous identification of these conspicuously large cholinergic synaptic contacts and the characteristic postsynaptic SSC for which they are named has prompted numerous investigations into their distribution, source, function, and pathology. Yet despite the detailed morphologic and physiologic information generated by many neuroscientists, it is humbling to consider (a) the incrementally slow trajectory by which our understanding of this enigmatic synapse has grown and (b) that as yet there is no definitive and fully functional hypothesis regarding their distribution, their postsynaptic subcellular machinery, their contribution to motor control and behavior, and their regulation/dysregulation in health and disease.

Recently, we have learned the most elementary effect of C-boutons on α-MN *f-I* gain during static intracellular current injection occurs via dramatic reductions in the strength of the action potential AHP (Miles et al., 2007), which is mediated by postsynaptic small conductance Ca^2+^-activated K^+^ (SK) channels (Deardorff et al., 2013). However, the mystery of the C-bouton and its cholinergic effects on MN biophysical properties and integrative capabilities is by no means solved, as has been suggested (Frank, 2009). Using an isolated spinal cord preparation, Miles et al. (2007) demonstrate a putative role for C-boutons in ensuring appropriate levels of motor output during drug induced fictive locomotion. But complexity arises upon behavioral assessment of adult mice with selective genetic inactivation of C-bouton synaptic inputs, which during locomotion exhibit normal flexor–extensor alternation and normal EMG amplitude. Motor deficits in these mice primarily manifest during *high-output* tasks such as swimming (Zagoraiou et al., 2009). These data convincingly implicate C-boutons in the task-dependent regulation of α-MN excitability via reduction of outward K^+^ currents, but questions remain regarding (a) the functional impact of C-bouton input during different behaviors, (b) the manner in which C-bouton activity is modulated to match motor demands, (c) the expression of abnormal force generation as well as spasticity, rigidity, or tremor as a consequence of C-bouton dysfunction, and (d) the mechanism of interaction between underlying acetylcholine receptors (AChRs) and K^+^ channels.

To aid in the development of new *in vivo* and *in vitro* experimental strategies to answer these and related questions, this review details our current understanding of the cellular, synaptic, and genetic properties that underlie C-bouton function and proposes a hitherto unexplored mechanism for the cholinergic modification of α-MN excitability. It should be noted that the title of this review is intended to reflect and pay homage to the many dedicated and careful neuroscientists who have undertaken MN synaptological investigations over the years. This review will therefore also provide historical perspective on the foundational advances in our understanding of this complex and elusive, yet important, synapse. Neuroscientists have spent 50+ years at the C-bouton swimming against the tide. Significant progress has been slow and hard fought. And though we are a long way from shore, we must remember – as our murine colleagues have demonstrated – without C-boutons we cannot swim at all.

## THE C-BOUTON SIGNALING ENSEMBLE: A CONTEMPORARY VIEW OF A CLASSIC SYNAPSE

**FIGURE 1:**
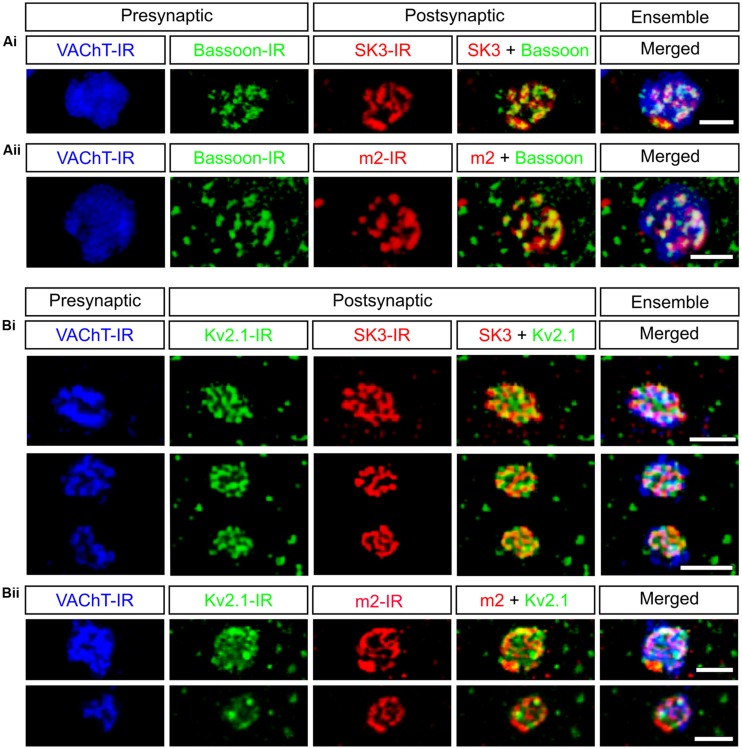
**C-bouton synaptic sites contain a complex signaling ensemble.** Presynaptic bassoon-IR and postsynaptic SK3-IR and m2-IR share a striking subsynaptic fenestrated appearance within the C-bouton. All images are small confocal stacks (3 × 1 μm Z-stacks) of *en face* C-boutons, indicated with VAChT-IR (blue), on rat lumbar α-motoneurons. **(A)** Presynaptic active zone protein bassoon (green) is aligned with postsynaptic ion channels SK3 (**Ai**, red) and m2 receptors (**Aii**, red). **(B)** Kv2.1-IR (green) intercalates with SK3-IR (Bi, red) and m2-IR **(Bii)**, “filling in” the C-bouton postsynaptic membrane. Scale bars are 2.0 μm.

**FIGURE 2:**
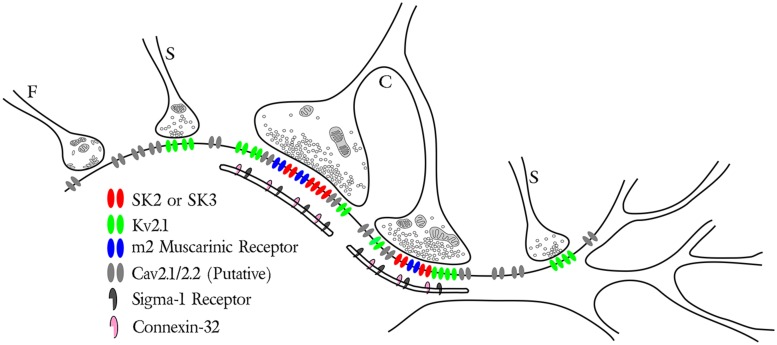
**Synaptic distribution of specific ion channels and receptors on soma and proximal dendrites of motoneurons.** The schematic illustrates three types of motoneuron presynaptic boutons including the glycinergic/GABAergic F-type, glutamatergic S-type and cholinergic C-type with its associated postsynaptic subsurface cistern. Note the specific localization of m2 muscarinic receptors (blue) with SK channels (red) and Kv2.1 channels (green) postsynaptic to the C-bouton. Small Kv2.1 clusters are also found postsynaptic to some S-type synapses (see Muennich and Fyffe, 2004). The P/Q- and N-type Ca^2+^ channels Cav2.1/2.2 (light gray) are illustrated throughout the membrane, although the precise subcellular localization of this channel is currently unknown. Both connexin 32 (pink) and the sigma-1 receptor (dark gray) are specifically associated with the C-bouton subsurface cistern.

We are riding the crest of a wave. With the turn of the century and the application of advanced morphologic analyses, cellular neurophysiology, and selective genetic perturbations, we have built a decidedly robust picture of C-bouton form and function. C-boutons are an essential piece of an integrated control system set to regulate α-MN activity through a complex anatomical substrate: a signaling ensemble (**Figures 1** and **2**) precisely organized for highly nuanced orchestration of somatic K^+^ currents.

### PRECISE ANATOMICAL LOCALIZATION AND ORGANIZATION OF SIGNALING COMPONENTS: AN ENSEMBLE OF APPOSED PROTEINS AND MOSAIC MEMBRANE DOMAINS

C-type synaptic sites comprise three closely apposed membranous domains (**Figure 2**), spanning a breadth of <25 nm, and across which the distribution of synaptic and signaling proteins are precisely regulated. Clear and consistent immunohistochemical data demonstrate membrane clusters of α-MN Kv2.1 channels, SK2/3 channels, and m2 receptors directly apposing C-bouton presynaptic terminals (Skinner et al., 1999; Hellstrom et al., 2003; Muennich and Fyffe, 2004; Wilson et al., 2004; Deardorff et al., 2013). When visualized under high resolution, these SK2/3 channel and m2 receptor clusters are composed of an intricate, non-uniform aggregation of smaller “threadlike” structures that are woven together and closely approximate/appose C-bouton pre-synaptic vesicle release sites, which are enriched with bassoon (A. S. Deardorff, S. H. Romer, R. E. W. Fyffe, unpublished; see **Figure 1**). Beneath the postsynaptic membrane, in α-MN SSCs, the gap junction protein connexin32 shows a similar threadlike distribution pattern (Yamamoto et al., 1990, 1991; Zampieri et al., 2014), indicating that connexin32, SK channels/m2 receptors, and transmitter release machinery are precisely aligned across the three membranous domains. Kv2.1 channels appear to “fill in” the remaining postsynaptic α-MN membrane surface not occupied by SK channels or m2 receptors. The demarcated postsynaptic area, therefore, is a highly structured and mosaic domain of interdigitating clusters of Kv2.1 channels and co-localized SK2/3 channels and m2 receptors. The orderly, stacked apposition of proteins on the cisternal, postsynaptic, and presynaptic membranes as well as the spatial interdigitation of distinct channel and receptor clusters demonstrates a coordinated and specific signaling organization across all membranous domains at C-bouton synaptic sites.

### ADDITIONAL SIGNALING COMPONENTS

Additional studies have revealed, to varying levels of specificity, other signaling components that characterize the C-bouton ensemble. Certain elements, although identified within one or another membranous or cytoplasmic domain, are not well defined in regard to specific subdomain organization nor anatomic relation to other molecular components. In this category, C-bouton synaptic terminals express a range of exocytotic proteins consistent with those necessary for fast transmitter release (Hellstrom et al., 1999), are highly associated with presynaptic P2X_7_ purinergic receptor immunoreactivity (~90% of C-boutons; Deng and Fyffe, 2004), and may also express presynaptic nicotinic acetylcholine receptors (nAChRs; Khan et al., 2003). In addition, the α-MN SSC is highly enriched with S1Rs (Mavlyutov et al., 2010), and with closely associated neuregulin-1 (NG1) immunoreactivity (Gallart-Palau et al., 2014). Indole-*N*-methyl transferase (INMT), an enzyme that converts tryptamine into the S1R ligand dimethytryptamine (DMT), is also present in close proximity to S1Rs at C-bouton postsynaptic sites (Mavlyutov et al., 2012), but the extent to which S1Rs, themselves, are diffusely distributed within the entire cisternal membrane or co-localize/interdigitate with the well-characterized connexin32 immunoreactivity is not described.

The subcellular organization of Ca^2+^ sources necessary for SK channel activation also remains poorly characterized. However, α-MN SK2/3 channels require high voltage activated (HVA) N- and P/Q-type Ca^2+^ currents to generate the AHP (Viana et al., 1993; Umemiya and Berger, 1994; Bayliss et al., 1995; Li and Bennett, 2007), and SK channels typically couple to their Ca^2+^ source(s) by <200 nm (Fakler and Adelman, 2008; Jones and Stuart, 2013). Internally, SSCs may amplify or shape these Ca^2+^ signals via RyRs or connexin32, as they do in other cell types (see discussion Section “Subsurface Cisternae and the Generation of an Isolated Ca^2+^ Signal”). We, therefore, expect some proportion of HVA Ca^2+^ channels and RyRs to localize to the C-bouton postsynaptic membrane and/or to the associated SSC (**Figure 2**). In support, Wilson et al. (2004) provide evidence that P/Q-type Ca^2+^ channels are diffusely spread throughout the α-MN somatic membrane. By inference, some proportion must then appose C-boutons. The presence of N-type Ca^2+^ channels on α-MNs, however, has only been demonstrated physiologically (Carlin et al., 2000; Wilson et al., 2004).

### THE CREST OF A WAVE

The unique aggregation of cytoplasmic and membrane bound pre- and postsynaptic proteins that constitute the C-bouton signaling ensemble provides mechanistic insight into the cholinergic modulation of α-MN firing rate and has advanced new research at a comparatively faster pace than that of many other α-MN synaptic inputs. While uncertainties still confound our arrival at a “simple” molecular mechanism governing C-bouton synaptic function, experiments in other cell systems can help push us forward against the tide. Further exploration of this complex synapse is clearly necessitated. However, we must first review other salient features of the C-bouton system.

## MOMENTS AND MILESTONES: ULTRASTRUCTURE

**FIGURE 3:**
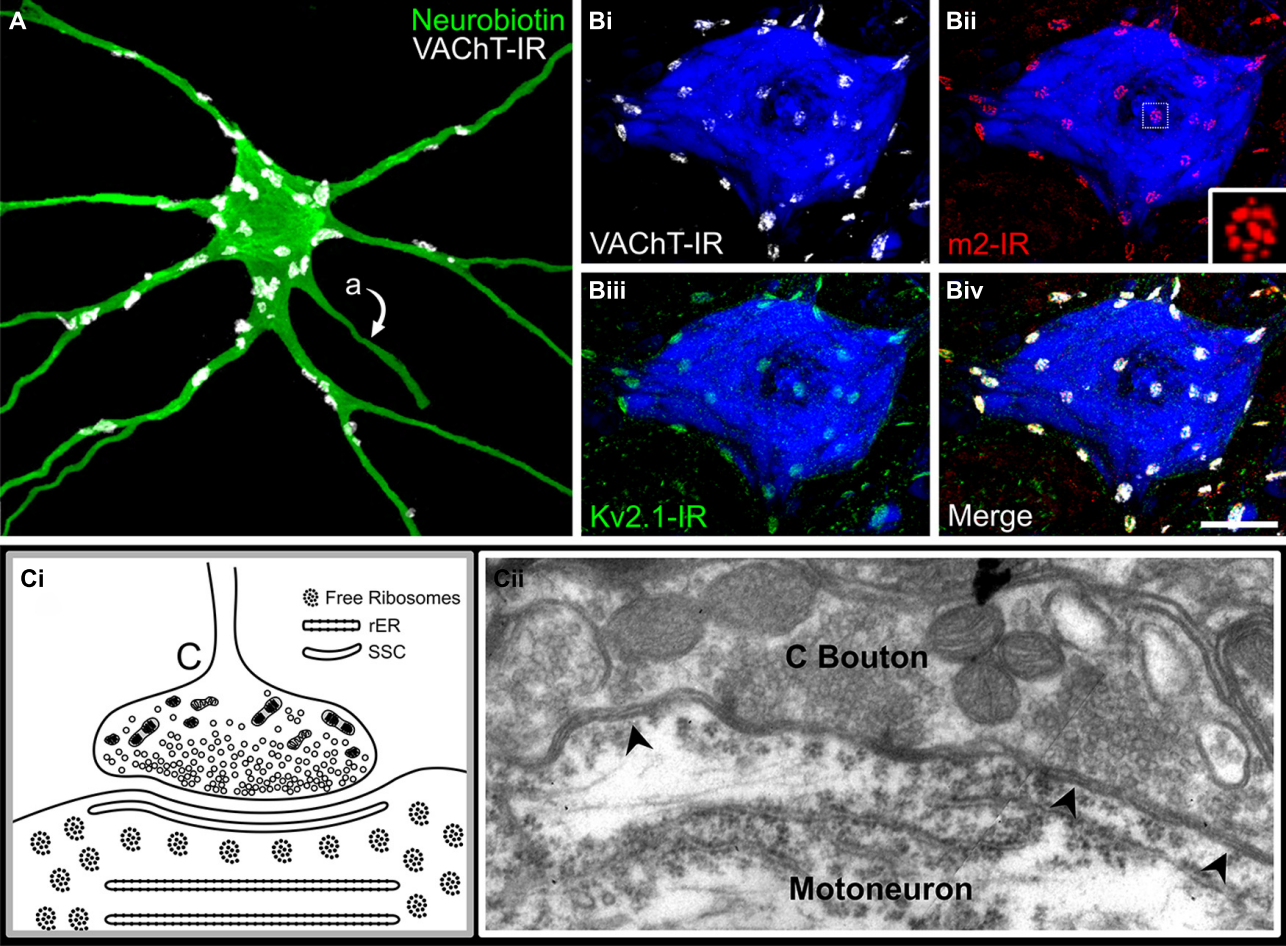
**The C-bouton synapse on mammalian α-motoneurons. (A)** C-bouton synapses on intracellularly labeled and reconstructed adult rat lumbar α-MN are revealed by VAChT-IR (white). Large C-boutons densely innervate the soma and proximal dendrites of α-MNs but are absent from more distal locations. Also note that C-boutons are not located on motoneuron axons (indicated by “a”). **(B)** C-boutons, indicated by VAChT-IR (**Bi,iv**, white), are presynaptic to the muscarinic m2 receptor (**Bii,iv**, red) and large Kv2.1 clusters (**Biii,iv**, green). Note that m2 receptor immunoreactivity on the α-MN soma and proximal dendrites localize exclusively to C-bouton postsynaptic sites. **(Bii)** Inset shows subsynaptic fenestrated distribution of m2-IR. Images are confocal stacks of 12 × 1 μm Z-stacks with nissl stain (blue) to label adult rat neuronal somata. Scale bar is 20 μm. **(C)** Diagrammatic representation and electron micrograph of C-bouton ultrastructure in an adult rat. **(Ci)** Diagram illustrates densely packed, clear spherical or pleomorphic vesicles and abundant mitochondria. Closely apposed to the postsynaptic membrane is a 10–15 nm wide subsurface cistern (SSC) that is continuous with several lamellae of underlying rough endoplasmic reticulum (rER). Free ribosomal rosettes are typically visible in the subsynaptic region. **(Cii)** Electron micrograph of C-bouton synapse on an α-MN soma. Arrowheads indicate a SSC extending the entire appositional length of the bouton. Note key features present in electron micrograph illustrated in diagram **(Ci)**.

Pioneering EM investigations (Wyckoff and Young, 1956) provided accurate anatomical description and categorization of the structurally diverse presynaptic terminals contacting spinal α-MNs, and in general, most authors still conform to the descriptive abbreviations (S-, F-, C-, T-, and M-Boutons) introduced by Bodian (1966a,b) and Conradi (1969a). (An additional bouton type, the P bouton, makes presynaptic connections with specific excitatory boutons in contact with the MN surface and may form triadic arrangements; Conradi, 1969a; Fyffe and Light, 1984). Those boutons Conradi classified as “C-type” are defined by and named for a signature 10–15 nm thick postsynaptic SSC (“C-type” for cistern): a broad, flat disc of smooth endoplasmic reticulum juxtaposed a mere 5–8 nm below the postsynaptic membrane and spanning the length of the apposing presynaptic terminal (**Figure 3**; Conradi, 1969a). The SSC is continuous with several lamellae of rough endoplasmic reticulum oriented in parallel with the cell membrane and frequently observed alongside free ribosomal rosettes in the subcisternal cytoplasm (**Figure 3**). Across a particularly narrow synaptic cleft (3–8 nm; see discussion Davidoff and Irintchev, 1986), the C-boutons themselves contain a dense cytoplasmic matrix of glycogen particles and neurofilaments tightly packed with 25–55 nm (diameter) clear spherical/pleomorphic vesicles, abundant mitochondria, and occasionally a small number of large dense core vesicles intermingled therein (**Figure 3**; Bodian, 1966a,b; Conradi, 1969a; McLaughlin, 1972b; Hamos and King, 1980). Notably, several authors (Rosenbluth, 1962; Bodian, 1966a,b; Charlton and Gray, 1966; Van Harreveld and Khattab, 1967) identified these unique and prominent boutons prior to Conradi’s (1969a) classic and thorough description of their synaptic ultrastructure – which remains the gold standard for their identification.

C-boutons are among the largest of α-MN somatic and proximal dendritic synaptic inputs, ranging in size from 3 to 8 μm in the cat (Conradi, 1969a; McLaughlin, 1972b; Conradi et al., 1979a), 3–6 μm in the primate (Bodian, 1966a,b), 3–5 μm in the opossum (Hamos and King, 1980), 3–6 μm in the human (Pullen, 1992), and 1–8 μm in the rodent (Alvarez et al., 1999). But despite their conspicuous size, they lack quintessential active zone ultrastructure, i.e., pronounced paramembraneous densities and associated pools of readily releasable vesicles (Bodian, 1966a,b; Conradi, 1969a; McLaughlin, 1972b; Bernstein and Bernstein, 1976), prompting early speculation that vesicle release occurs across the entire synaptic interface (McLaughlin, 1972b). However, small presynaptic dense projections and local vesicle aggregations have been subsequently described (Hamos and King, 1980; Connaughton et al., 1986; Davidoff and Irintchev, 1986), and are particularly pronounced in non-osmicated tissue stained with E-PTA (Pullen, 1988a) or uranyl acetate and lead citrate (Schroder, 1979). These observations are commonly accepted evidence for *specific* synaptic vesicle release sites. Supporting this notion, C-boutons express discrete *punctae* of the active zone specific protein bassoon rather than diffuse expression throughout the presynaptic membrane (A. S. Deardorff, S. H. Romer, R. E. W. Fyffe, unpublished; see **Figure 1**). Moreover, bassoon immunoreactivity precisely overlies postsynaptic SK channels and m2 receptors even though traditional postsynaptic densities are not typically observed under EM. The physiologic advantage of this characteristically atypical and peculiarly subtle active zone architecture, however, is not yet fully understood, and may be further complicated by interspecies variability (see Pullen, 1988a).

C-boutons are ubiquitous and highly specific to somatic α-MNs and have been identified on α-MN somata and proximal dendrites in all mammalian species studied thus far (see Yamamoto et al., 1991 for references). Detailed analyses of γ-MNs (Lagerback, 1985; Lagerback et al., 1986; Destombes et al., 1992), autonomic MNs (Mawe et al., 1986; Leedy et al., 1988), spinal interneurons (Johnson and Sears, 1988), and Renshaw cells (Lagerback and Ronnevi, 1982; Alvarez and Fyffe, 2007) confirm these cells lack C-type synaptic inputs. C-boutons, when properly identified (see discussion Section “Moments and Milestones: Transmitter Content”), are thus a useful anatomical criterion to distinguish somatic α-MNs in the brain and spinal cord (Conradi, 1969a; Pullen, 1988b; Deng and Fyffe, 2004; Muennich and Fyffe, 2004; Deardorff et al., 2013). Although there have been no extensive three-dimensional analyses of the total number of C-boutons per α-MN, our, and other, observations suggest on the order of 30–70 such contacts per cell (McLaughlin, 1972b; Hamos and King, 1980; Brannstrom, 1993; Brannstrom and Kellerth, 1998), and in general, there are a greater number of C-bouton synaptic contacts on large α-MNs innervating fast twitch muscle fibers, with this difference not simply due to the larger available somatic/dendritic surface area (Conradi et al., 1979a,b; Kellerth et al., 1979, 1983; Hellstrom et al., 2003). It should be noted, the features of C-boutons present on somatic α-MNs in ocular motor nuclei vary from those in the spinal cord and other brainstem motor nuclei. Specifically, C-boutons have been ultrastructurally identified (Tredici et al., 1976) and α-MN SSCs express connexin32 (Yamamoto et al., 1991), but no large VAChT-IR synaptic contacts (Hellstrom et al., 2003) nor m2 receptors are present (Vilaro et al., 1992; Hellstrom et al., 2003).

## MOMENTS AND MILESTONES: TRANSMITTER CONTENT

Correlative light-electron microscopic analysis of ChAT-IR confirmed C-boutons are cholinergic (Houser et al., 1983; Connaughton et al., 1986; Li et al., 1995), a suggestion first made decades prior with ultrastructural acetylcholinesterase (AChE) histochemistry (Lewis and Shute, 1966), which alone is not sufficient for cholinergic classification (Fibiger, 1982; Satoh et al., 1983; Sakamoto et al., 1985; Davidoff and Irintchev, 1986; Nagy et al., 1993). In support, VAChT is highly associated with small clear synaptic vesicles in the C-bouton presynaptic terminal (Gilmor et al., 1996) and there is a strong association throughout the brainstem and spinal cord between large ChAT-IR synaptic boutons on α-MNs and SSCs immunolabeled for connexin32 (Nagy et al., 1993). Immunoreactivity for the cholinergic markers ChAT or VAChT, combined with anatomical criteria such as bouton size and location, therefore makes C-boutons easily identifiable in adult/neonatal histologic sections (**Figure 3**; Barber et al., 1984; Phelps et al., 1984; Nagy et al., 1993; Hellstrom et al., 2003; Wilson et al., 2004; Zagoraiou et al., 2009; Alvarez et al., 2011; Deardorff et al., 2013). However, this approach should be applied with caution, as a small subset of cholinergic S-type terminals arising from recurrent α-MN axon collaterals and contacting α-MN somata may approximate C-boutons in size (Cullheim et al., 1977; Lagerback et al., 1981). Definitive confirmation of C-bouton phenotype requires ultrastructural verification of the C-bouton specific “cisternal signature” or alternatively – when systematically surveying an adequate sample of α-MNs under EM is unrealistic – light level co-localization of cholinergic makers with C-bouton specific pre- and/or postsynaptic proteins (see Section “The C-bouton Signaling Ensemble: A Contemporary View of a Classic Synapse”).

## MOMENTS AND MILESTONES: DISSECTING THE C-BOUTON CIRCUITRY

Unlike so many α-MN synaptic inputs, for which the neurons of origin are identifiable anatomically and physiologically (Jankowska and Lindstrom, 1972; Jankowska and Roberts, 1972a,b; Brown et al., 1981; Brown, 1983; Fyffe, 1991a,b; Burke and Glenn, 1996; Bui et al., 2003), the neuronal source of C-boutons has been elusive. Early *in vivo* lesion studies demonstrated that C-boutons do not degenerate following dorsal root section (Conradi, 1969b; McLaughlin, 1972a; Bodian, 1975); spinal cord hemisection/transection (McLaughlin, 1972c; Bodian, 1975; Pullen and Sears, 1978, 1983), or cortical ablation (Bodian, 1975). Neither are they labeled by injection of retrograde tracers into dorsal roots (Ralston and Ralston, 1979), nor intracellular staining of Ia afferents (Brown and Fyffe, 1978; Conradi et al., 1983; Fyffe and Light, 1984), Ib afferents (Brown and Fyffe, 1979), group II afferents (Fyffe, 1979), hair follicle afferents (Maxwell et al., 1982), or axons innervating cutaneous mechanoreceptors (Brown et al., 1978, 1980, 1981; Bannatyne et al., 1984; Maxwell et al., 1984). Similarly, intracellular labeling of α-MNs showed C-boutons do not arise from α-MN axon collaterals (Lagerback et al., 1981), which is corroborated by differential protein expression in C-type synapses versus cholinergic terminals in the Renshaw cell area (see Section “The C-bouton Signaling Ensemble: A Contemporary View of a Classic Synapse;” Hellstrom et al., 1999; Deng and Fyffe, 2004).

Though these data collectively indicate the intraspinal derivation of C-boutons, no investigator to date has intracellularly labeled a cholinergic spinal interneuron and traced its axon to an α-MN C-type synaptic contact *in vivo* or *in vitro*; the definitive test for synaptic connectivity. Advanced molecular labeling techniques, however, have very convincingly demonstrated that C-boutons arise from cholinergic V0-embryonic (V0_C_) interneurons identifiable transcriptionally and phenotypically by the expression of the V0-specific homeobox protein Dbx1, the paired-like homeodomain transcription factor Pitx2, and the cholinergic proteins ChAT or VAChT (Miles et al., 2007; Zagoraiou et al., 2009). (For complete information on V0 cell ontogeny, we refer the reader to studies by Moran-Rivard et al. (2001), Pierani et al. (2001), and Lanuza et al. (2004) as well as the review by Arber (2012)). V0_C_ interneurons correspond to a known population of cholinergic partition cells (Barber et al., 1984; Phelps et al., 1984; Arvidsson et al., 1997) located lateral to the central canal in Rexed’s lamina X and medial lamina VII (Miles et al., 2007; Zagoraiou et al., 2009). They can be subdivided into ipsilateral and bilateral projecting subpopulations and span several segments rostral and caudal to their innervated motor pools (Stepien et al., 2010). Cholinergic partition cells, C-type synaptic boutons, and the “signaling ensemble” appear early in postnatal development, and are well established by approximately 1 month of age (Phelps et al., 1984; Wetts and Vaughn, 2001; Wilson et al., 2004).

The specific placement of V0_C_ interneurons within segmental spinal circuitry is not fully characterized [see preliminary circuit diagrams in Zagoraiou et al. (2009) and Witts et al. (2014)]. Preliminary analysis of V0_C_ connectivity demonstrates V0_C_ interneurons receive synaptic input from several sources, including descending serotonergic pathways, local and/or descending VGluT2 projections, inhibitory interneurons (e.g., V2b cells), lamina II/III nociceptive interneurons, and non-proprioceptive primary mechanosensors (Zagoraiou et al., 2009; Witts et al., 2014; Zampieri et al., 2014; Zhang et al., 2014). Each V0_C_ cell sends divergent axonal projections to several α-MNs of the same or functionally equivalent motor pools and avoids α-MNs innervating antagonist muscles (Stepien et al., 2010). Numerous *en passant* synaptic varicosities arising from a single V0_C_ axon contact the soma and proximal dendrites of a one or more α-MNs, which in turn receive convergent input from several V0_C_ cells (Stepien et al., 2010). Although the precise levels of convergence/divergence are unknown, this pattern of connectivity establishes a large number of release sites from each presynaptic axon onto the α-MN, likely reflecting a high probability of transmitter release and contributing to a high safety factor for strong cholinergic neuromodulation (e.g., Walmsley et al., 1998).

Recent work shows V0_C_ interneurons also project numerous *small* synaptic contacts onto V1-derived IaINs (Siembab et al., 2010). These synapses are morphologically dissimilar to C-boutons (Siembab et al., 2010), and their postsynaptic effects are currently unknown. Still, it is intriguing to consider that V0_C_ interneurons project to the only two neuronal types (α-MNS and IaINs) in the ventral horn known to receive both recurrent inhibition and group Ia excitatory drive. Whether V0_C_ interneurons, like Renshaw cells, send parallel projections to α-MNs and the “corresponding” IaINs (i.e., those with the same Ia connections; Hultborn et al., 1971a,b,c) has yet to be elucidated. Nevertheless, these data provide further insight into segmental motor circuitry and prompt new questions into both circuit function and synaptic specificity of the V0_C_ neuronal class.

## MOMENTS AND MILESTONES: AHP, SK, AND MOTOR UNIT TYPE

**FIGURE 4:**
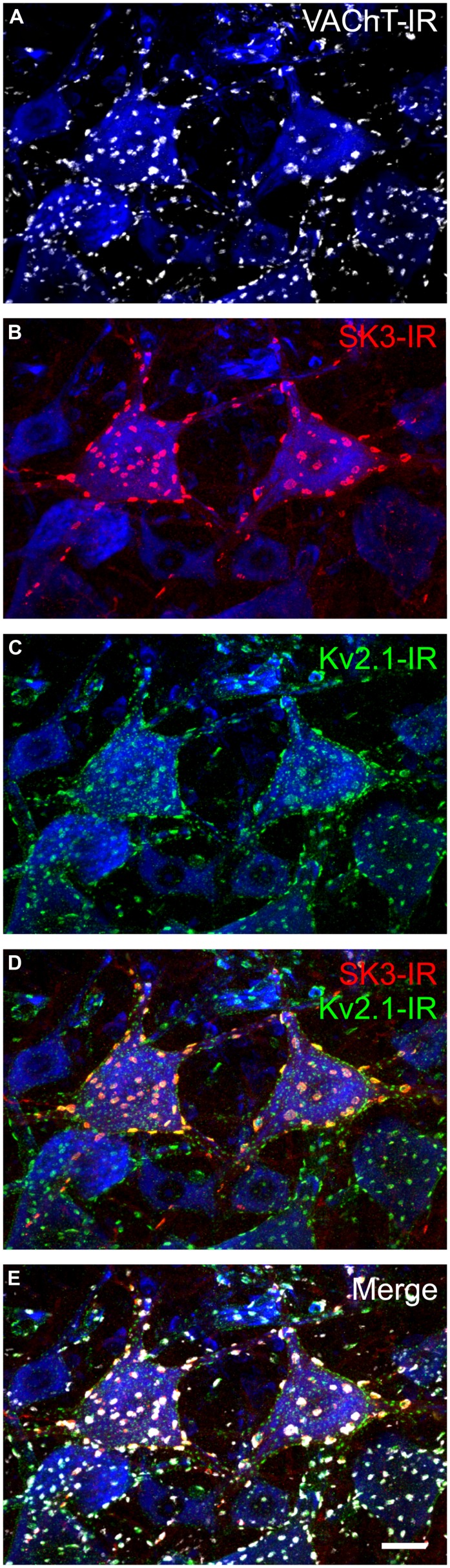
**The potassium ion channel SK3 is part of the C-bouton signaling ensemble in a subset of α-motoneurons.** Images are confocal stacks of 26 × 1 μm Z-stacks with nissl stain (blue) to label rat lumbar neuronal somata. Scale bar is 20 μm. **(A)** VAChT-IR (white) C-boutons form synapses onto all rat lumbar α-MNs on the soma and proximal dendrites. **(B)** SK3-IR (red) located within surface membrane of a subset of α-MNs in large distinct clusters. In rodents, SK3 channels, having slower intrinsic activation and deactivation kinetics than SK2 channels (Xia et al., 1998), are preferentially expressed in small, presumably S-type, α-MNs with long duration and large amplitude mAHP currents (Deardorff et al., 2013). **(C)** Large and small Kv2.1-IR (green) clusters are located within the surface membrane of all α-MNs. **(D,E)** The large SK3-IR and Kv2.1-IR clusters colocalize within the surface membrane of α-MNs and are apposed to VAChT-IR C-boutons.

**FIGURE 5:**
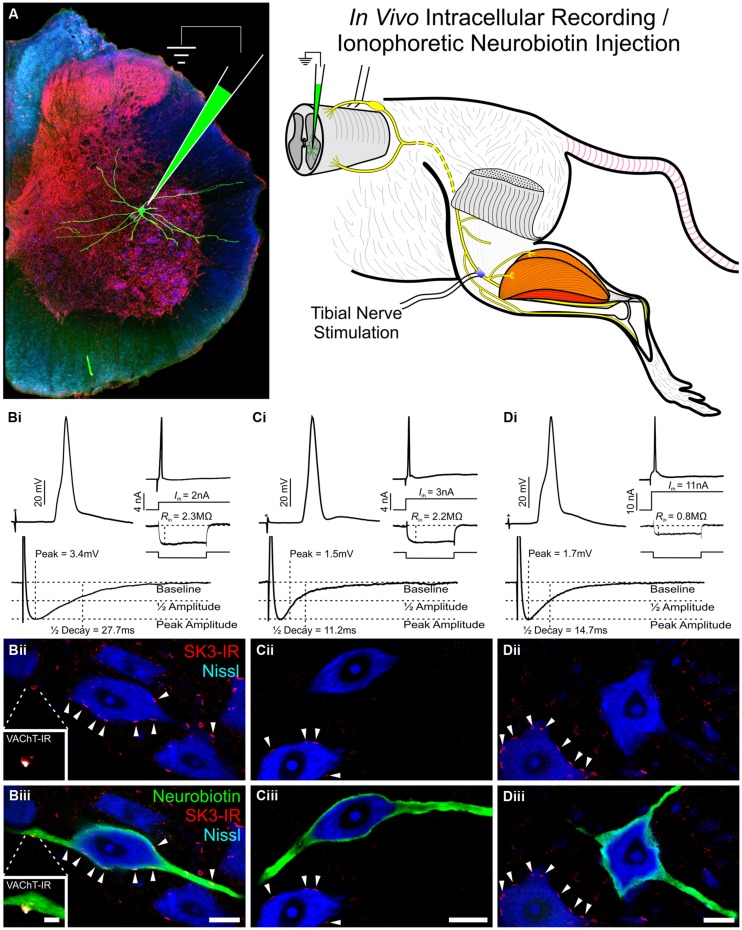
**Subset of rat lumbar α-motoneurons with SK3-IR have significantly longer AHP 1/2 decay time and increased amplitude. Data shown is review of previous study reported by Deardorff et al. (2013). (A)** Diagrammatic representation of experimental paradigms. In an adult *in vivo* rat preparation, tibial α-MNs, identified by antidromic activation of the tibial nerve, were penetrated with a sharp recording electrode. Neuronal electrical properties were recorded and neurons were filled with neurobiotin (green) for *post hoc* identification. Spinal cord tissue was harvested and processed for SK3-IR. **(B–D)** Neuronal electrical properties are of α-MNs depicted in micrographs below. Asterisk (*) denotes stimulus artifact. Micrographs are single optical confocal sections through the soma of intracellularly labeled α-MNs (green) processed for SK3-IR (red) and the general neuronal stain nissl (Blue). Scale bars are 20 μm. **(B)** SK3-IR (+) (**Bii** and **Biii** arrowheads) α-MNs have long duration and large amplitude AHP, low rheobase, and high input resistance. Micrograph insets show VAChT-IR (White) C-bouton in apposition to an SK3-IR (+) cluster. Inset scale bar is 5 μm. **(C,D)** SK3-IR (-) α-MNs have short duration and small amplitude AHPs. However, even among these SK3-IR (-) cells, rheobase and input resistance show high variance along the continuum of α-MN properties. Please note the nearby SK3-IR (+) cells (**C,Dii,iii** arrowheads).

Early *in vivo* use of the SK channel blocker, apamin, established that SK channels are uniquely responsible for generating α-MN AHP currents (Zhang and Krnjevic, 1987). *In vitro* investigation subsequently confirmed these findings (Viana et al., 1993; Lape and Nistri, 2000), and showed that α-MN SK currents are reduced following m2 receptor activation at C-bouton synaptic sites (Lape and Nistri, 2000; Miles et al., 2007). Consistent with these electrophysiological data, our lab has recently shown that not only are SK channels highly enriched in the C-bouton postsynaptic membrane (Deardorff et al., 2013), but individual α-MNs express a variable complement of SK2 and SK3 channel isoforms consistent with observed co-variability in α-MN size and AHP duration (Deardorff et al., 2013). In the rodent, all α-MNs express SK2, but SK3 expression is markedly heterogeneous and cell-type-specific (**Figure 4**) varying in intensity from negligible (<2× background) to modest (2 to 3× background) to strong (>3× background) between individual α-MNs in a single tissue section. SK3 channels, which have a longer deactivation time constant than SK2 (Xia et al., 1998), are *only* expressed (with SK2) at C-bouton postsynaptic sites in smaller α-MNs with longer duration/larger amplitude AHPs (**Figure 5**). Conversely, larger α-MNs with significantly shorter duration/smaller amplitude AHPs express only SK2 (with little or no SK3-IR; **Figure 5**).

SK3-expressing α-MNs share other physiological properties predictive of S-type MNs (i.e., slower conduction velocity, lower rheobase, and higher input resistance; Deardorff et al., 2013). SK3-IR within the signaling ensemble can therefore provide “brush stroke” differentiation of rodent α-MNs along their physiological spectrum, and is a useful tool for histologic analysis of α-MN subtypes in development and disease (Brownstone and Magown, 2013). Altogether these data strongly indicate that the relative proportion of SK2/SK3 isoforms and the channel cluster size and density regulates AHP duration and amplitude, and the variability of these proportions accounts, in part, for the fact that AHP properties are continuous variables across a population of α-MNs (Deardorff et al., 2013). SK channel expression may, therefore, explain the “speed match” between AHP duration of a given α-MN and the contractile speed of its innervated muscle fibers (Bakels and Kernell, 1993; Gardiner, 1993). However, critical additional factors include the source and amplitude of the necessary Ca^2+^ signal, the coupling of these signals to the SK channels and, potentially, the presence/absence of *I_h_* currents (Gustafsson and Pinter, 1985). Nevertheless, differential SK channel expression at the C-bouton undoubtedly contributes to α-MN input–output gain across the spectrum of α-MN subtypes by regulating AHP properties.

## SWIMMING FORWARD: A MECHANISM FOR CHOLINERGIC MODULATION

We return now to the crest of our wave. The constancy of form and the intricacy of protein expression imply a fundamental logic to C-bouton organization and engagement during motor activity. Here, we assert the signaling ensemble is built around an organizing principle (i.e., the SSC) that allows for the generation of isolated Ca^2+^ signals at multiple sites on the soma. From this starting point, our intent here is to swim forward toward the synthesis of a comprehensive mechanistic hypothesis for the cholinergic modulation of α-MN firing rate. We base our rationale in the now recognized functional requirement for C-boutons in “swimming” (Zagoraiou et al., 2009), in the observation that cholinergic C-bouton function is *not required* for regular locomotion (Zagoraiou et al., 2009), and in the probable interactions of the key components of the C-bouton signaling ensemble (**Figures 1** and **2**), most of which are known to generate, regulate, or be regulated by local intracellular Ca^2+^.

**FIGURE 6:**
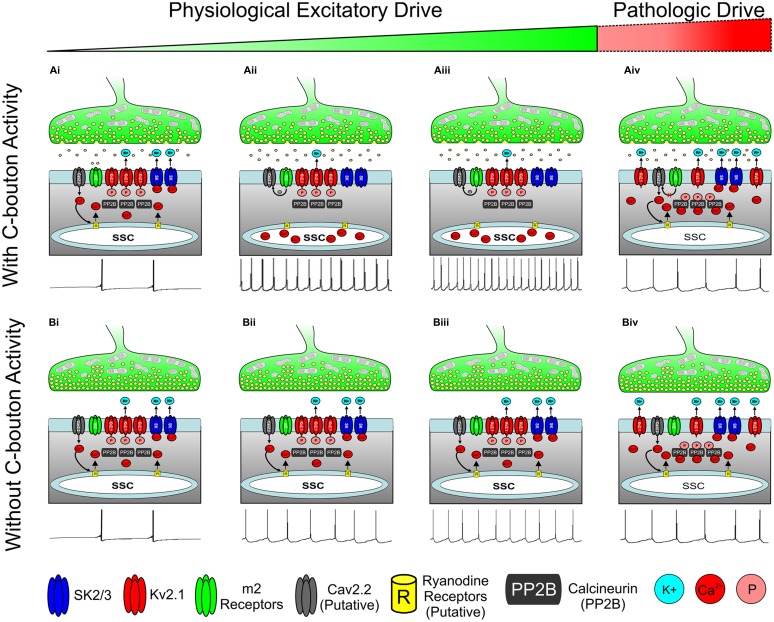
**Hypothesis for state dependent regulation of motoneuron activity through the C-Bouton signaling ensemble. (A)** C-boutons increase motoneuron firing frequency along a widow of the α-MN activity spectrum. **(Ai)** With low or transient physiological drive, m2 activation is not likely to mediate an effect on AHP duration or firing rate. **(Aii,iii)** As excitatory drive increases, persistent m2 receptor activation inhibits local Ca_V_ channels through a G_i_/G_o_ coupled pathway, preventing both the SK channel activation and Kv2.1 dephosphorylation. Thus, outward K^+^ current is reduced and neuronal firing rate is increased (relative to **Bii** and **Biii**) as illustrated with spike train below. **(Aiv)** m2-mediated effects on Ca_V_ channels are negated by prolonged or repeated membrane depolarization (Hille, 1994) as may occur during extremely strong or pathologic excitatory drive. Here, Ca^2+^ influx through N-type calcium channels activates SK channels to generate AHP and to dephosphorylate Kv2.1 to increase outward K^+^ current and reduce firing frequency, as illustrated with spike train below. **(Bi–iii)** As excitatory drive increases without C-bouton activity, the N-type Ca^2+^ influx activates SK channels to generate AHP. Thus, the outward K^+^ current maintains a lower firing frequency than in corresponding images in A. Spike trains illustrated below. **(Biv)** As in **(Aiv)**, during prolonged or pathologic excitatory drive, N-type Ca^2+^ influx results in both SK channel activation and Kv2.1 dephosphorylation, thereby increasing outward K^+^ current and homeostatically decreasing firing rate, illustrated with spike train below. All spike trains depicted in this figure are added for illustrative purposes only and do not represent electrophysiological recordings or computer simulations.

Although C-boutons may boost recruitment gain, as proposed elsewhere (Zagoraiou et al., 2009; Brownstone and Magown, 2013), we propose that the cholinergic modulation produced by C-boutons is highly task-dependent and will be maximal only during the moderate to strong physiological drive necessary for high-output motor tasks like swimming (Zagoraiou et al., 2009; **Figure 6**). The mechanism we suggest accounts for the minimal appreciable requirements and effects observed during conditions of low and/or transient drive, which are appropriate for spinal reflexes and/or low-output tasks such as walking (Zagoraiou et al., 2009; **Figures 6Ai,Bi**). We extend this notion further to conditions of extremely powerful physiological (or pathological) drive, during which time any effects of C-bouton activity on firing rate are negated by the molecular dynamics and kinetics of the respective m2 receptors and SK/Kv channels (**Figures 6Aiv,Biv**). That is, while the cumulative, combined effects of these isolated Ca^2+^ signals on specific AHP and delayed rectifier K^+^ currents are likely to be quite significant throughout the α-MN activity spectrum, the functional impact of the C-bouton circuitry is only observed when imposed upon a restricted window of moderate to strong excitatory drive. We believe our synthesis, which is primarily based on interpretation of disparate datasets, will promote testable hypotheses. Elements of this synthetic approach are considered in the following sections.

### SUBSURFACE CISTERNAE AND THE GENERATION OF AN ISOLATED Ca^2+^ SIGNAL

It is widely accepted that neuronal SSCs function as an intracellular Ca^2+^ store with multiple roles in Ca^2+^ homeostasis and mobilization (see Yamamoto et al., 1991 and Fuchs et al., 2014 for references). Indeed, Henkart et al. (1976) proposed that SSCs “are designed to release Ca^2+^ into the cytoplasm with whatever further effects this might produce.” SSCs serve also as a physical diffusion barrier that spatially and functionally restricts this Ca^2+^ signal from those originating in other cellular compartments and, during increased cellular activity, act as a Ca^2+^ sink to rapidly absorb and shuttle free Ca^2+^ from the cisternal microdomain (Yamamoto et al., 1990, 1991; Fuchs et al., 2014). Ca^2+^ release by RyR-rich SSCs serve, in part, to activate nearby SK channels in cochlear hair cells, which share some synaptic similarities with C-boutons (Evans et al., 2000; Lioudyno et al., 2004; Grant et al., 2006), and in sympathetic ganglion cells (Akita and Kuba, 2000). RyR release of Ca^2+^ may also result in an increase in nearby Kv2.1 channel conductances, via Ca^2+^-dependent dephosphorylation pathways, as it does in hippocampal and cortical pyramidal cells (Du et al., 1998; Antonucci et al., 2001; Misonou et al., 2005). Moreover, vesicles observed budding from the cytoplasmic surface of SSCs in cochlear hair cells and α-MNs are thought to be involved in removal of excess free Ca^2+^ from the subsynaptic cytoplasm (Yamamoto et al., 1991; Fuchs et al., 2014). In light of these factors, the SSC itself is highly indicative that the functional regulation of the C-bouton signaling ensemble (which includes SK and Kv2.1 channels) occurs through precise control of an isolated Ca^2+^ microdomain, the mechanistic underpinnings of which are considered below.

### INVOLVEMENT OF THE SIGNALING ENSEMBLE WITH THE ISOLATED Ca^2+^ SIGNAL

The unique aggregation of cellular elements at C-bouton synaptic sites and their coordinated regulation *by* and/or *of* the isolated Ca^2+^ signal enables exquisite control over α-MN K^+^ currents. Consider first the generation of the α-MN AHP. Membrane bound N- and P/Q-type Ca^2+^ currents necessary for α-MN SK channel activation (see “Additional Signaling Components”) generate this Ca^2+^ signal, which is isolated and shaped by the SSC. The AHP currents influence repetitive discharge properties of α-MNs, in part, via reductions in the variability in the interspike interval, the slope of the *f-I* relation, and the maximal rate of primary-range firing (Kernell, 2006; Brownstone and Magown, 2013).

A primary effect of m2 receptor activation by C-bouton synapses is a reduction of the AHP (Lape and Nistri, 2000; Miles et al., 2007). Though their signaling pathway(s) in α-MNs are undefined, m2 receptors typically exert their effects by inhibiting N-type Ca^2+^ channels, as observed in sympathetic ganglion (Hille, 1994; Herlitze et al., 1996; Shapiro et al., 1999), cortical pyramidal (Stewart et al., 1999), neostriatal (Howe and Surmeier, 1995), and basal forebrain neurons (Allen and Brown, 1993). Ca^2+^ influx through these channels is required for activation of SK channels and dictates the number of SK channels that open. N-type channel blockade by m2 receptors is usually mediated by G_i/o_ protein coupled βγ subunits, which cause a depolarizing shift in the voltage dependence of channel activation (Hille, 1994; Herlitze et al., 1996; Ikeda, 1996; Jeong and Ikeda, 1999; Shapiro et al., 1999) and is negated by strong or repeated membrane depolarization (Hille, 1994).

The m2/cholinergic effect exerted by active C-boutons is quite simple and intuitive at this level: preventing N-type Ca^2+^ influx (which is largely triggered by synaptically evoked action potentials) from activating SK channels during moderate to strong physiologic drive of the MNs (**Figures 6Aii,iii,Bii,iii**). This would be consistent with *observed* reduction of the AHP and enhanced α-MN excitability when m2 receptors are, presumably, activated during swimming or other tasks requiring high motor output (e.g., **Figures 6Aii,Aiii**; Miles et al., 2007; Zagoraiou et al., 2009). This “upstream” mechanism of AHP modulation will have a minimal appreciable effect on individual AHPs and α-MN firing rate during low levels of physiologic drive causing transient or “subprimary” range firing (Manuel et al., 2009; Turkin et al., 2010), due to the *physiological* triggering of SK channel activation by a short duration, suprathreshold stimulus (i.e., an action potential) occurring at intervals that may be longer than the duration of the AHP itself (**Figures 6Ai,Bi**). This may account for observations that C-bouton function is not required for regular locomotion (Zagoraiou et al., 2009). Moreover during powerful and/or pathologic excitatory drive the m2-mediated diminution of N-type channel activity is negated (Hille, 1994), resulting in a break of the m2 generated effect and an increase in AHP size (**Figures 6Aiv,Biv**).

At high levels of excitatory drive we must also consider the results of modulation of other components of the signaling ensemble. Although the m2 mediated effect on AHP is significant in a particular physiological range, the *whole* microdomain has an important role in setting α-MN firing rate. With this in mind, the Ca^2+^ dependent generation of the AHP and its regulation by m2 receptor activation is one part of a coordinated series of molecular events that occur at the C-bouton, but is reliant on the complex interplay of other components in the signaling ensemble. For example, as excitatory drive increases how does the combinatorial contribution of SK and/or Kv2.1 change in the presence or absence of cholinergic input?

In the highly clustered configuration (typically) observed in hippocampal and cortical pyramidal cells, and α-MNs, Kv2.1 channels are phosphorylated and have a high activation and deactivation threshold and slow kinetics (Murakoshi et al., 1997; Misonou et al., 2004, 2005; Surmeier and Foehring, 2004; Mohapatra and Trimmer, 2006; Misonou, 2010). Interestingly, some investigators have postulated that clustered Kv2.1 channels serve primarily non-conducting functions (O’Connell et al., 2010; Fox et al., 2013); for the purposes of this discussion we will consider a more traditional role for the channels in αMNs. Importantly, upon prolonged/pathologic excitatory drive, Ca^2+^/calcineurin dependent dephosphorylation pathways (**Figures 6Aiv,Biv**) rapidly decluster Kv2.1 while simultaneously lowering its activation and deactivation threshold and accelerating its kinetics (Surmeier and Foehring, 2004; Park et al., 2006; Mohapatra et al., 2009). In α-MNs, prolonged excitatory drive causes rapid Kv2.1 channel declustering (Romer et al., 2014) by a Ca^2+^/calcineurin dependent mechanism (S. H. Romer, A. S. Deardorff, R. E. W. Fyffe, unpublished), though corresponding alterations in channel kinetics are uncharacterized.

Data from other cell types shows clustered Kv2 channels maintain steady state firing by regulating membrane potential during the interspike interval (Johnston et al., 2008; Guan et al., 2013; Liu and Bean, 2014), while declustered/dephosphorylated Kv2 channels serve to homeostatically lower firing rate (Surmeier and Foehring, 2004; Park et al., 2006; Mohapatra et al., 2009). In this way, Kv2 channels may increase or decrease cell excitability depending on the kinetics of channel activation (Liu and Bean, 2014). Brownstone et al. (2011) propose C-bouton activity during fictive locomotion (Miles et al., 2007; Zagoraiou et al., 2009) may contribute to steady state firing rates via the regulation of Kv2.1 phosphorylation and clustering. This is consistent with our hypothesis that m2-mediated inhibition of HVA-Ca^2+^ current prevents the activation of Ca^2+^/calcineurin dependent dephosphorylation pathways and thus maintains Kv2.1 clustering. However, if prolonged/pathologic excitatory drive causes large changes in intracellular Ca^2+^ sufficient to allow diffusion of Ca^2+^ from neighboring compartments, there would be rapid Kv2.1 channel declustering (Romer et al., 2014) by a Ca^2+^/calcineurin dependent mechanism (S. H. Romer, A. S. Deardorff, R. E. W. Fyffe, unpublished), negating the influence of C-boutons.

Several other components of this complex signaling ensemble likely serve to fine tune the efficacy of neuromodulation. Presynaptic nAChRs and P2X_7_ receptors may provide an additional regulatory mechanism for synaptic transmission, particularly if ATP is co-released with ACh as it is at other central and peripheral cholinergic synapses (Burnstock et al., 1997), and cisternal S1Rs are known to reduce the sensitivity of m2 receptors to ACh (Walker and Bourguignon, 1990; Kim et al., 2010). Altogether, we suggest the C-bouton signaling ensemble is a highly integrated system, organized around an anatomically segregated Ca^2+^ microdomain, for precise and nuanced regulation of cell firing. Moreover, it has a built-in fail-safe mechanism against excitotoxicity, in that this strategically organized ensemble can both be driven by, or override, the synaptic circuitry of the C-bouton.

### AN ALTERNATIVE MECHANISM

Others have suggested, based on muscarine’s minimal effect on global α-MN Ca^2+^ currents, that m2 receptor activation results in the direct blockade of α-MN SK channels (Miles et al., 2007; Witts et al., 2014). In support of their view, the direct phosphorylation of SK channels by protein kinase A (PKA) and casein kinase 2 (CK2) can, respectively, cause channel internalization (Kohler et al., 1996; Ren et al., 2006; Fakler and Adelman, 2008; Faber, 2009) and reduced Ca^2+^ sensitivity (Bildl et al., 2004; Allen et al., 2007). Moreover, neurotransmitter-initiated signaling cascades have been shown to modulate SK channel gating through CK2- or protein kinase C (PKC)-mediated phosphorylation (Maingret et al., 2008; Buchanan et al., 2010; Giessel and Sabatini, 2010). Although m2 receptors typically inhibit protein kinase activity, they can activate phosphorylation pathways in smooth muscle (Zhou et al., 2003). Therefore it is possible the direct phosphorylation of SK channels by protein kinases could provide an alternate mechanism through which m2 receptors reduce the AHP in α-MNs.

However, evidence that N- and P/Q-type Ca^2+^ channels are diffusely distributed throughout the α-MN somatic membrane (Wilson et al., 2004), and that α-MN SSCs function as Ca^2+^ diffusion barriers indicates that m2 receptor activation need only inhibit those α-MN Ca_V_ channels located within or very near to the C-bouton postsynaptic membrane to exert an effect on the AHP. In this case, m2 influence over the signaling ensemble would be masked in studies of global Ca^2+^ currents. The activation of CK2- or PKC-mediated phosphorylation would also be a novel finding for neuronal m2 receptors, necessitating future studies characterizing this undescribed signaling pathway. Moreover, such a mechanism would act as a binary switch, turning on and off AHP when necessary and not requiring an elaborate signaling ensemble nor the SSC. Our hypothesis, however, of a signaling ensemble organized around fine control of a Ca^2+^ micro-signaling domain is capable of highly nuanced and graded modulation of outward K^+^ current.

## C-BOUTONS IN HUMAN HEALTH AND DISEASE

Dynamic reorganization of C-boutons and components of the postsynaptic signaling ensemble has been noted in a variety of pathologic conditions and in conditions of altered excitability (Saxena et al., 2013; Romer et al., 2014; Witts et al., 2014). The bulk of the data has thus far been obtained in animal models, and there is no consensus on whether C-bouton plasticity in these conditions is compensatory or pathologic. In part, the uncertainty results from the diversity of disease/injury models that affect C-boutons and the complexity of the signaling ensemble.

Analysis of effects on C-bouton structure in models of amyotrophic lateral sclerosis (ALS), spinal cord injury, and peripheral nerve injury demonstrate diverse and sometimes conflicting reports. In ALS, there has been interest in potential neuroprotective roles for C-boutons and this view is bolstered by studies that show an early increase in C-bouton size (Pullen and Athanasiou, 2009; Herron and Miles, 2012; Saxena et al., 2013); however, diminished C-bouton and V0c interneuronal ChAT/VAChT content (Nagao et al., 1998; Casas et al., 2013) and S1R expression (Casas et al., 2013; see Witts et al., 2014) have also been observed in similar murine models of the disease. The structural changes in animal models may also reflect a propensity for C-bouton reorganization to occur first in larger, less excitable, and more vulnerable α-MNs (Saxena et al., 2013), and the changes may be more pronounced in males (Herron and Miles, 2012). There is minimal data from autopsied human spinal cord from ALS patients, mostly from late stages of the disease, showing continued presence of C-boutons on degeneration-resistant sphinteric α-MNs (Pullen, 1992). Additionally, the duration of the AHP in human MNs is possibly related to disease progression (i.e., an initial shortening followed by prolongation; Piotrkiewicz and Hausmanowa-Petrusewicz, 2011).

C-bouton organization is affected by both spinal cord and peripheral nerve injury, which generally appear to cause transient or persistent loss of and/or disconnection of C-boutons from α-MNs and changes in expression and localization of SK, HCN, and Kv2.1 channels (Kerns and Hinsman, 1973; Sumner, 1975; Alvarez et al., 2011; Romer et al., 2012, 2014). These specific changes may account for some, but not all, of the physiological changes that have been observed (Kuno et al., 1974a,b; Cope et al., 1986; Bichler et al., 2007a,b; Bullinger et al., 2011; Prather et al., 2011), including altered post-spike AHP duration and repetitive firing properties (Kuno et al., 1974a; Gustafsson and Pinter, 1984).

The significance of C-bouton plasticity remains uncertain. After injury, the specific loss or disconnection could lead to postsynaptic receptors (m2) becoming constitutively active, analogous to observations made of the serotoninergic system (Fouad et al., 2010; Kong et al., 2010, 2011; Murray et al., 2010, 2011; Hultborn et al., 2013), but this has not been explored. Given the high vulnerability of large, F-type α-MNS in ALS, it would be interesting to determine if the graded expression of SK channel isoforms will promote new testable hypotheses regarding disease pathogenesis and C-bouton mediated compensatory adjustments (Brownstone and Magown, 2013; Deardorff et al., 2013).

## CONCLUSION

Multiple neuromodulatory systems and a myriad of ion channels are available for the task dependent regulation of MN excitability. The serotonergic system, for example, originates in the brainstem raphe nucleus, provides extensive synaptic input onto α-MN dendrites (Alvarez et al., 1998) and is strongly linked to both behavioral and pathologic alterations of persistent inward Ca^2+^ currents (Li and Bennett, 2003, 2007; Heckmann et al., 2005; Brownstone, 2006; Heckman et al., 2008; Norton et al., 2008; Powers et al., 2008). While numerous studies have focused on *inward* current modulation, the state dependent regulation of α-MN *outward* current has only recently been investigated (see Manuel et al., 2012). New evidence has shown that a cholinergic modulatory system originating from spinal interneurons (V0_C_ interneurons), and contributing dense synaptic coverage to α-MN somata, modulates the strength of motor output via reductions in α-MN outward K^+^ current (Miles et al., 2007; Zagoraiou et al., 2009). It is interesting to consider that while serotonin increases MN excitability by *amplifying inward* current, acetylcholine does so by *reducing outward* current. The dynamic interplay of these two different, but rather synergistic, systems endows the CNS with remarkable control over MN output, and the interaction between the AHP and L-type Ca^2+^ currents responsible for PIC may be a critical factor in regulating α-MN firing properties (Manuel et al., 2014).

Here, we illustrate large, cholinergic presynaptic terminals, termed C-boutons (Conradi, 1969a), are important modulatory loci for state-dependent alterations in MN repetitive firing, largely mediating their effects through a unique and highly specialized signaling ensemble organized for the state-dependent regulation of outward K^+^ currents. To effectively manipulate signal transduction at C-bouton synaptic sites may be critical in the development of new therapeutic interventions for a variety of devastating neurological conditions. However, advances in patient care will first require a complete understanding of both the transduction mechanisms, as well as which cases (if any) C-bouton synaptic reorganization and/or alterations in α-MN AHP (and other intrinsic α-MN properties) contribute to disease pathology or, alternatively, maintain α-MN viability.

## Conflict of Interest Statement

The authors declare that the research was conducted in the absence of any commercial or financial relationships that could be construed as a potential conflict of interest.

## ABBREVIATIONS

AHP: afterhyperpolarization
ChAT: choline acetyltransferase
IaIN: Ia inhibitory interneuron
IR: immunoreactivity
m2 receptor: type 2 muscarinic acetylcholine receptor
MN: motoneuron
RyR: ryanodine receptors
S1R: sigma-1 receptors
SK: small conductance calcium-activated potassium channel
SSC: subsurface cistern
VAChT: vesicular acetylcholine transporter
VGluT: vesicular glutamate transporter.
